# Surgical management of cecal volvulus using endoscopic reduction as a bridge to elective laparoscopic surgery: a three-case series

**DOI:** 10.1093/jscr/rjag971

**Published:** 2026-10-30

**Authors:** Kaita Suzuki, Koshiro Harata, Ryo Taguchi, Rena Saito, Nobuaki Kagohashi, Ryosuke Niwamoto, Motoki Futamata, Tsuyoshi Saito, Shuji Koide, Takeyasu Katada, Futoshi Teranishi, Shuji Takiguchi

**Affiliations:** Department of Gastroenterological Surgery, Toyokawa City Hospital, 23 Noji, Yawata-cho,Toyokawa, Aichi 442-8561, Japan; Department of Gastroenterological Surgery, Toyokawa City Hospital, 23 Noji, Yawata-cho,Toyokawa, Aichi 442-8561, Japan; Department of Gastroenterology, Toyokawa City Hospital, 23 Noji, Yawata-cho, Toyokawa, Aichi 442-8561, Japan; Department of Gastroenterological Surgery, Toyokawa City Hospital, 23 Noji, Yawata-cho,Toyokawa, Aichi 442-8561, Japan; Department of Gastroenterological Surgery, Toyokawa City Hospital, 23 Noji, Yawata-cho,Toyokawa, Aichi 442-8561, Japan; Department of Gastroenterological Surgery, Toyokawa City Hospital, 23 Noji, Yawata-cho,Toyokawa, Aichi 442-8561, Japan; Department of Gastroenterological Surgery, Toyokawa City Hospital, 23 Noji, Yawata-cho,Toyokawa, Aichi 442-8561, Japan; Department of Gastroenterological Surgery, Toyokawa City Hospital, 23 Noji, Yawata-cho,Toyokawa, Aichi 442-8561, Japan; Department of Gastroenterological Surgery, Toyokawa City Hospital, 23 Noji, Yawata-cho,Toyokawa, Aichi 442-8561, Japan; Department of Gastroenterological Surgery, Toyokawa City Hospital, 23 Noji, Yawata-cho,Toyokawa, Aichi 442-8561, Japan; Department of Gastroenterological Surgery, Toyokawa City Hospital, 23 Noji, Yawata-cho,Toyokawa, Aichi 442-8561, Japan; Department of Gastroenterological Surgery, Graduate School of Medical Sciences, Nagoya City University, 1 Kawasumi, Mizuho-cho, Mizuho-ku, Nagoya, Aichi 467-8601, Japan

**Keywords:** cecal volvulus, endoscopic reduction, bridge to surgery, colon volvulus, elective laparoscopic surgery

## Abstract

Cecal volvulus is a rare cause of bowel obstruction, typically managed by emergency surgery. Endoscopic reduction is generally not recommended due to its low success rate and risk of perforation. However, emergency surgery often requires laparotomy and may be associated with substantial surgical invasiveness. To reduce surgical invasiveness and avoid the risks associated with emergency surgery, we adopted a bridge-to-surgery strategy using endoscopic reduction followed by elective laparoscopic resection, limited to patients without suspected necrosis or perforation. We report three cases. Endoscopic reduction was successful in two patients, allowing subsequent elective laparoscopic surgery. In contrast, reduction failed in one patient, who underwent emergency surgery, during which cecal necrosis and perforation were identified. The patient subsequently died postoperatively. This case series discusses the potential role, indications, and current limitations of endoscopic reduction as a bridge to surgery in cecal volvulus.

## Introduction

Cecal volvulus accounts for approximately 1–1.5% of all cases of intestinal obstruction and is the second most common type of colonic volvulus. However, its incidence is low, ranging from 2.7 to 7.1 cases per million population annually [1]. Surgical intervention with bowel resection is generally recommended. In contrast, endoscopic reduction is not routinely performed as a first-line treatment because of its relatively low success rate, risk of procedure-related perforation, and high recurrence rate [2].

However, emergency surgery often requires laparotomy because of marked bowel dilatation, resulting in greater surgical invasiveness [3, 4]. In selected patients, successful endoscopic reduction can achieve bowel decompression, allowing elective laparoscopic surgery while avoiding the potential risks associated with emergency surgery. Based on this concept, we attempted endoscopic reduction only in selected patients without peritoneal signs, elevated lactate levels, or computed tomography (CT) findings strongly suggestive of bowel necrosis or perforation.

We report three patients with cecal volvulus who underwent this bridge-to-surgery strategy. To the best of our knowledge, published reports describing endoscopic reduction as a bridge to surgery for cecal volvulus remain extremely limited. Our series included one patient in whom endoscopic reduction failed and emergency surgery was required, allowing us to discuss not only the potential clinical usefulness of this strategy but also its indications and limitations.

## Case presentation

### Case 1

A 42-year-old man presented to our emergency department with upper abdominal pain that had developed 1 hour earlier. On admission, he was hemodynamically stable. Physical examination revealed abdominal distension without tenderness or peritoneal signs. Laboratory tests revealed a white blood cell (WBC) count of 6700/μL, a C-reactive protein (CRP) level of 0.06 mg/dL, and an arterial lactate level of 1.9 mmol/L. Contrast-enhanced CT demonstrated marked cecal dilatation with slightly decreased enhancement of a portion of the bowel wall. No intraperitoneal free air, ascites, whirl sign, or closed-loop formation was identified (Fig. 1).

**Figure 1:**
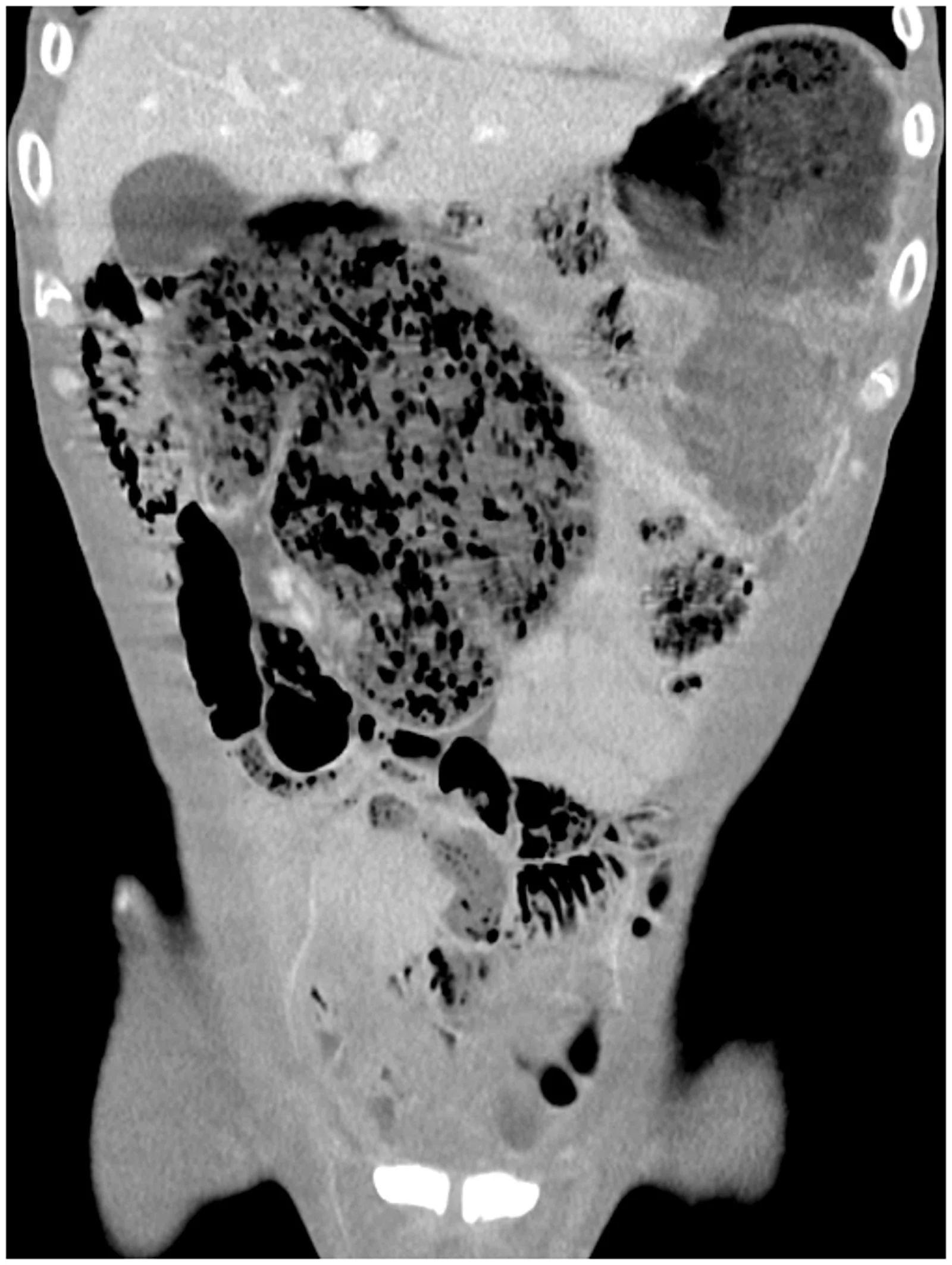
Contrast-enhanced CT showing a markedly dilated cecum with slightly decreased bowel wall enhancement.

Because bowel necrosis or perforation was considered unlikely, endoscopic reduction was performed. The volvulus was easily reduced without endoscopic evidence of mucosal ischemia. After reduction, abdominal pain and distension promptly improved. Until surgery, nutritional support consisted of parenteral nutrition and an oral liquid nutritional formula. Elective laparoscopic ileocecal resection was performed on hospital day 9, and the patient was discharged on postoperative day 14.

### Case 2

A 77-year-old woman presented to our emergency department with epigastric pain that had persisted for 12 hours. On admission, she was hemodynamically stable. Physical examination revealed mild abdominal distension without tenderness or peritoneal signs. Laboratory tests revealed a WBC count of 7200/μL, a CRP level of 0.76 mg/dL, and a lactate level of 0.7 mmol/L. Contrast-enhanced CT demonstrated a dilated cecum with preserved bowel wall enhancement. No intraperitoneal free air, ascites, whirl sign, or closed-loop formation was identified (Fig. 2).

**Figure 2:**
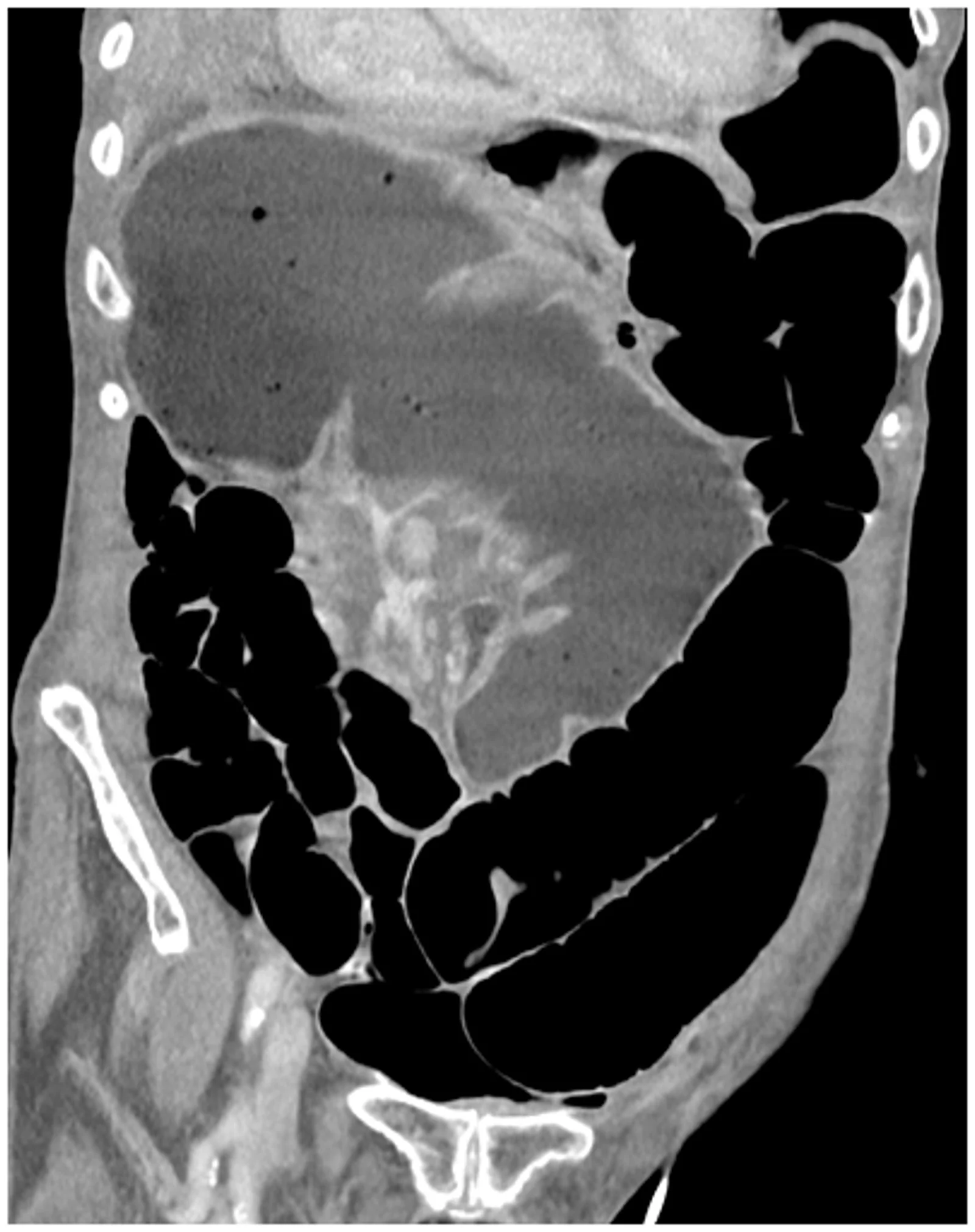
Contrast-enhanced CT showing a dilated cecum in the upper abdomen with preserved bowel wall enhancement.

Because bowel necrosis or perforation was considered unlikely, endoscopic reduction was performed. The volvulus was easily reduced without endoscopic evidence of mucosal ischemia. Abdominal distension resolved within a few hours after reduction. Until surgery, the patient received parenteral nutrition and an oral liquid nutritional formula. Elective laparoscopic ileocecal resection was performed on hospital day 6, and the patient was discharged on postoperative day 8.

### Case 3

An 86-year-old man presented to our emergency department after experiencing anorexia and vomiting for 5 days without abdominal pain. On admission, he was hemodynamically stable. Physical examination revealed abdominal distension without tenderness or peritoneal signs. Laboratory tests revealed a WBC count of 7700/μL, a CRP level of 7.44 mg/dL, and a lactate level of 0.8 mmol/L. Contrast-enhanced CT demonstrated a markedly dilated ileocecal segment with a whirl sign, closed-loop formation, and slight decreased enhancement of part of the bowel wall. A small amount of ascites was present, whereas no intraperitoneal free air was detected (Fig. 3).

**Figure 3:**
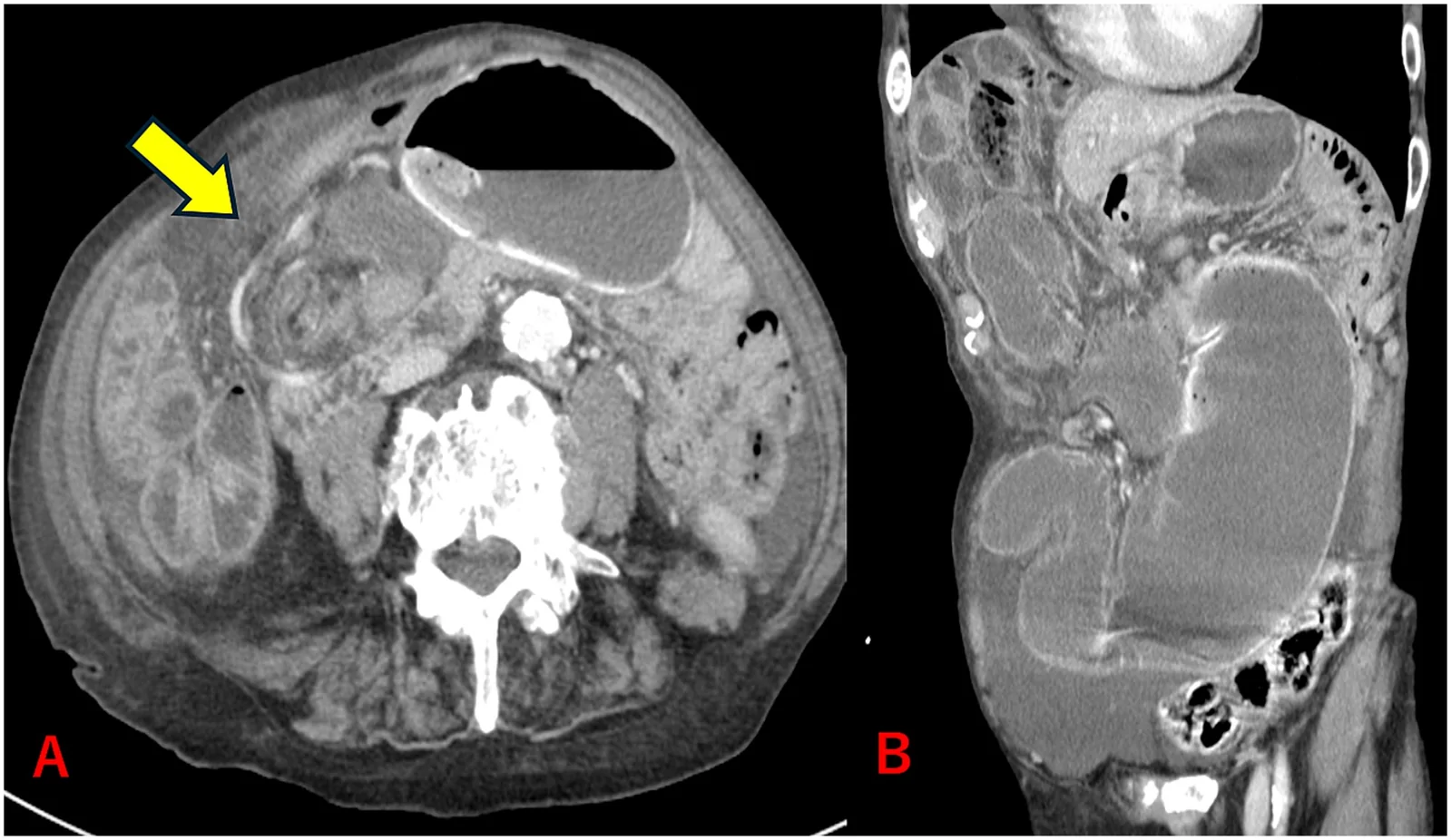
Contrast-enhanced CT findings in case 3. (A) Axial CT image showing a whirl sign. (B) Coronal CT image showing a markedly dilated cecum with a small amount of ascites and slightly decreased bowel wall enhancement.

Although the laboratory and CT findings did not strongly suggest bowel necrosis or perforation, the slight decrease in bowel wall enhancement and the relatively indolent clinical course suggested mild ischemia rather than irreversible bowel necrosis. Therefore, endoscopic reduction was attempted. However, the endoscope could not be advanced beyond the stenotic segment, and newly developed mucosal ischemia was observed during the procedure. Endoscopic reduction was abandoned, and emergency surgery was immediately performed.

Laparotomy revealed cecal perforation with generalized peritonitis and a localized area of cecal necrosis (Fig. 4). Ileocecal resection with diverting ileostomy was performed. Despite intensive postoperative management, septic shock progressed, and the patient died on postoperative day 1.

**Figure 4:**
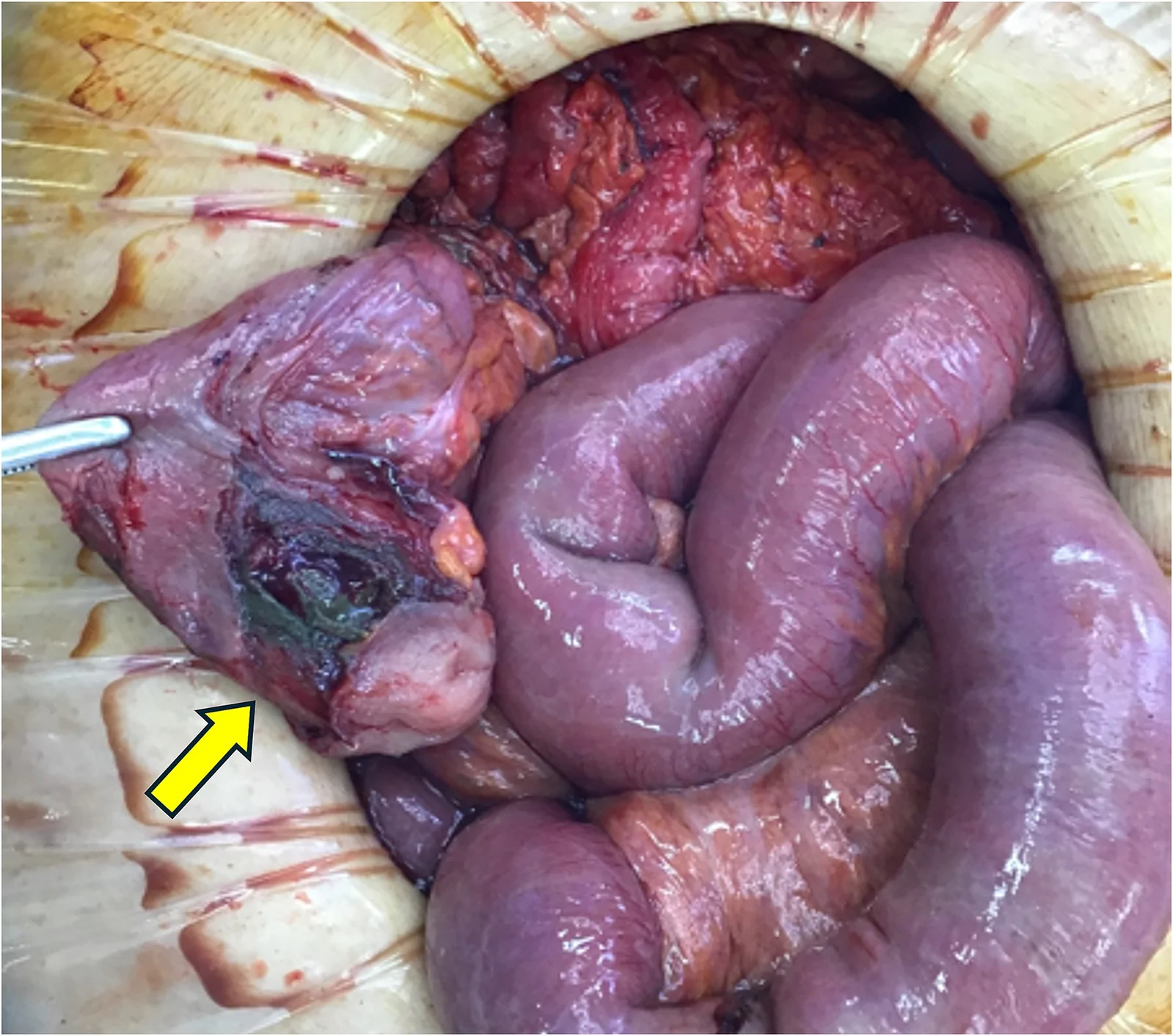
Intraoperative findings showing focal bowel wall necrosis and cecal perforation.

## Discussion

According to the Clinical Practice Guidelines of the American Society of Colon and Rectal Surgeons (ASCRS), endoscopic reduction is generally not recommended for cecal volvulus because of its low success rate, risk of procedure-related perforation, and high recurrence rate [2]. However, cecal volvulus is an uncommon disease, and the available evidence remains limited because of the small number of reported cases. Furthermore, most studies on which the current recommendations are based were published before the widespread adoption of laparoscopic surgery. Consequently, the strategy of elective laparoscopic resection following endoscopic reduction has not been adequately investigated [3, 5–7]. Although several reports have described successful endoscopic reduction [7–10], published reports on the use of endoscopic reduction as a bridge to subsequent laparoscopic surgery remain extremely limited. The present case series therefore provides a novel perspective on this treatment strategy for cecal volvulus.

The principal advantage of endoscopic reduction as a bridge to surgery is that bowel decompression may permit elective laparoscopic resection. In our series, successful endoscopic reduction enabled elective laparoscopic ileocecal resection in two patients, thereby avoiding laparotomy. Furthermore, in older patients or those with severe comorbidities who are at high risk for emergency surgery, successful endoscopic reduction may allow optimization of the patient’s condition before definitive surgery. In addition, the availability of anesthesiologists and operating room staff may differ during nights or holidays among institutions, and elective surgery may allow treatment under a safer operative setting. Thus, incorporating elective surgery into the treatment strategy may enable more flexible clinical decision-making according to each institution’s medical resources and clinical circumstances.

A retrospective review of our three patients suggested that detailed CT assessment and symptom duration may influence the feasibility and safety of endoscopic reduction. CT assessment should focus on findings suggestive of bowel necrosis or perforation and on the volvulus configuration. Previous reports have described free air as suggestive of perforation, and findings such as decreased or absent bowel wall enhancement, pneumatosis intestinalis, and bowel wall thickening as suggestive of bowel ischemia. Ascites has also been reported as a nonspecific CT sign of severe volvulus [11]. In Case 3, ascites was observed on preoperative CT; however, no other findings strongly suggested bowel necrosis or perforation, and these conditions were considered unlikely. Despite this, cecal necrosis and perforation were identified intraoperatively. Although ascites alone is not definitive, its presence may warrant greater caution when considering endoscopic reduction. Delabrousse et al. classified cecal volvulus into three subtypes according to the mechanism of torsion: axial torsion, loop, and bascule types [12]. Axial torsion and loop types are frequently associated with a whirl sign, whereas the loop type is additionally characterized by closed-loop obstruction and is more likely to cause severe bowel ischemia. In contrast, the bascule type typically lacks both the whirl sign and closed-loop configuration and is generally associated with less severe vascular compromise. Based on these findings, endoscopic reduction may be more feasible in patients with the bascule type or mild axial torsion than in those with the loop type. In our series, neither the whirl sign nor closed-loop formation was observed in the two successfully treated patients, whereas both findings were present in the patient in whom endoscopic reduction failed, suggesting that this patient most likely had the loop type. The duration of symptoms may also be an important factor. The two successfully treated patients presented shortly after symptom onset, whereas the unsuccessful patient had experienced symptoms for more than five days. Prolonged bowel distension and poor nutritional status resulting from persistent intestinal obstruction may have increased bowel wall fragility, ultimately leading to perforation. Taken together, these findings may be useful in assessing the feasibility and safety of endoscopic reduction. However, because they are based on observations from a limited number of patients, they should not be regarded as established selection criteria.

This study has several limitations. Because cecal volvulus is a rare surgical emergency, prospective or large-scale studies are difficult to conduct, limiting the accumulation of high-quality evidence. In addition, considerable heterogeneity in disease severity and patient characteristics makes it difficult to establish uniform indications for treatment. Notably, although laboratory findings and CT findings did not strongly suggest bowel necrosis or perforation, intraoperative examination in Case 3 revealed cecal perforation and focal bowel wall necrosis. This discrepancy highlights the limitations of preoperative assessment of bowel viability, even when clinical, laboratory, and CT findings do not strongly suggest bowel necrosis. Consequently, serious complications, including perforation, cannot be completely avoided even when endoscopic reduction is attempted.

Taken together, this strategy should not be routinely applied but may be considered a treatment option in carefully selected patients. Based on our experience, endoscopic reduction as a bridge to surgery may be considered in patients without clinical, laboratory, or CT findings strongly suggestive of bowel necrosis or perforation, particularly those with a volvulus configuration considered favorable for reduction and early presentation after symptom onset. However, these considerations are based on limited experience and require further validation in additional cases.

## Conclusion

For cecal volvulus, a surgical management strategy incorporating endoscopic reduction as a bridge to elective laparoscopic surgery may represent a novel treatment option in highly selected patients. However, preoperative assessment has limitations, and serious adverse outcomes may still occur. This strategy remains exploratory at present, and its indications should be determined with caution.

## References

[1] Gingold D, Murrell Z. Management of colonic volvulus. Clin Colon Rectal Surg 2012;25:236–44. 10.1055/s-0032-1329535 PMC3577612 24294126

[2] Alavi K, Poylin V, Davids JS et al. The American Society of Colon and Rectal Surgeons clinical practice guidelines for the management of colonic volvulus and acute colonic pseudo-obstruction. Dis Colon Rectum 2021;64:1046–57. 10.1097/DCR.0000000000002159 34016826

[3] Swenson BR, Kwaan MR, Burkart NE et al. Colonic volvulus: presentation and management in metropolitan Minnesota, United States. Dis Colon Rectum 2012;55:444–9. 10.1097/DCR.0b013e3182404b3d 22426269

[4] Rout A, Pillai S. Caecal volvulus: high index of suspicion is the key to early diagnosis. J Surg Case Rep 2021;2021:rjab517. 10.1093/jscr/rjab517 PMC8634507 34868549

[5] Friedman JD, Odland MD, Bubrick MP. Experience with colonic volvulus. Dis Colon Rectum 1989;32:409–16. 10.1007/BF02563694 2629714

[6] Theuer C, Cheadle WG. Volvulus of the colon. Am Surg 1991;57:145–50. 2003701

[7] Renzulli P, Maurer CA, Netzer P et al. Preoperative colonoscopic derotation is beneficial in acute colonic volvulus. Dig Surg 2002;19:223–9. 10.1159/000064217 12119526

[8] Suzuki H, Yamamura T, Fujishiro M. Endoscopic detorsion using single-balloon endoscopy for cecal volvulus. Dig Endosc 2020;32:149. 10.1111/den.13558 31597202

[9] Mizuguchi Y, Mamada Y, Shimizu T et al. Cecal volvulus following elective laparoscopic cholecystectomy: a case report. J Nippon Med Sch 2016;83:172–6. 10.1272/jnms.83.172 27680486

[10] Sakamoto Y, Hiyoshi Y, Sakata K et al. Case of cecal volvulus successfully treated with endoscopic colopexy. Asian J Endosc Surg 2018;11:402–4. 10.1111/ases.12460 29322628

[11] Vandendries C, Jullès MC, Boulay-Coletta I et al. Diagnosis of colonic volvulus: findings on multidetector CT with three-dimensional reconstructions. Br J Radiol 2010;83:983–90. 10.1259/bjr/35714052 PMC3473716 20965909

[12] Delabrousse E, Sarliève P, Sailley N et al. Cecal volvulus: CT findings and correlation with pathophysiology. Emerg Radiol 2007;14:411–5. 10.1007/s10140-007-0647-4 17618472

